# The Association of Physical Function With Psychosocial Patient‐Reported Outcomes in People With Systemic Lupus Erythematosus

**DOI:** 10.1002/acr.80065

**Published:** 2026-05-03

**Authors:** Mrinalini Dey, Jessica Fitzpatrick, Maria Dall'Era, Charmayne Dunlop‐Thomas, Courtney Hoge, S. Sam Lim, Patricia P. Katz, Jinoos Yazdany, Laura C. Plantinga

**Affiliations:** ^1^ Kings’ College London London United Kingdom; ^2^ University of California San Francisco California; ^3^ Emory University Atlanta Georgia

## Abstract

**Objective:**

To evaluate how modifiable psychosocial factors and fatigue relate to physical functioning in patients with systemic lupus erythematosus (SLE).

**Methods:**

In this cross‐sectional study of two demographically distinct cohorts (Approaches to Positive, Patient‐Centered Experiences of Aging with Lupus [APPEAL] and California Lupus Epidemiology Study [CLUES]), perceived physical function was assessed using Patient‐Reported Outcomes Measurement Information System Physical Function Short Forms 12a (APPEAL) and 10a (CLUES). Fatigue, depression, and stress; coping efficacy and learned helplessness (APPEAL); and resilience and self‐efficacy (CLUES) were all assessed with validated questionnaires. We used multivariable linear regression analyses to assess associations between perceived physical function and outcomes, adjusting for age, sex, and race, then further for disease damage and activity.

**Results:**

APPEAL participants (N = 451) were primarily Black (82.5%); CLUES participants (N = 580) were predominantly Asian (29.3%), Hispanic (22.9%), and White (27.4%). Mean physical function T scores were lower in APPEAL (43.6) versus CLUES (47.2; *P* < 0.05). In both cohorts, higher fatigue, depression, and perceived stress were strongly associated with poorer physical function. Greater resilience (per +1 SD: β = 2.04, 95% confidence interval [CI] 0.57–3.50) and self‐efficacy (high vs low: β = 13.53, 95% CI 8.69–18.37) (both in CLUES) and better coping (per +1 SD: β = 3.15, 95% CI 2.39–3.91; in APPEAL) were associated with better physical function. In APPEAL, greater learned helplessness was associated with worse physical function (per +1SD: β = −3.88, 95% CI −4.62 to −3.14). Although adjusting further for disease damage and activity attenuated these associations, they remained statistically significant.

**Conclusion:**

Our results demonstrate associations of fatigue and psychosocial factors with physical functioning, reinforcing their relationship with overall health in SLE.

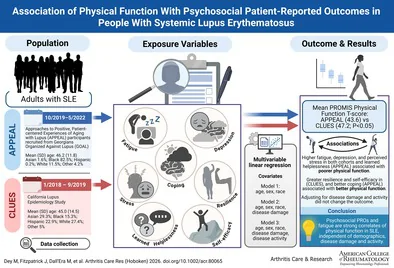

## INTRODUCTION

Physical disability is common among people living with systemic lupus erythematosus (SLE), which can impair quality of life, decrease social and work activity, and increase health care costs.^1^, ^2^ Approximately 30% of individuals with SLE are considered frail, whereas over half are estimated to have poor physical performance.^3^, ^4^ Limitations in basic and instrumental activities of daily living are seen in people living with SLE regardless of age, a pattern typically seen in older individuals in the general population.^5^ Functional decline in SLE is multifactorial, associated with higher disease activity, comorbidities such as depression, and difficulty working or socializing.^6^

**SIGNIFICANCE & INNOVATIONS**

- Physical function is a core outcome in systemic lupus erythematosus (SLE) and a major driver of disability; however, few studies have comprehensively evaluated how psychosocial factors are associated with functional status across diverse patient populations.
- This study represents one of the largest and most diverse investigations to date examining psychosocial factors and physical function in SLE, drawing on two well‐characterized cohorts with markedly different demographic and socioeconomic profiles.
- We have demonstrated that several patient‐reported outcomes, including fatigue, depression, perceived stress, coping, and self‐efficacy, are robustly and consistently associated with physical function, independent of demographics and SLE‐related damage and SLE activity.
- Our findings reinforce the importance of routinely incorporating patient‐reported outcomes into clinical practice, not only to capture patient experiences, which are shaped by the intersection between psychosocial factors and physical function, but also to inform assessment and guide holistic, patient‐centered interventions.

Patient‐reported outcome measures (PROMs), such as the Patient‐Reported Outcomes Measurement Information System (PROMIS) Physical Function assessments, offer valuable insights into people's lived experience of SLE.^7^ Psychosocial factors, as well as closely related perceptions of fatigue, which may otherwise be difficult to assess using traditional clinical disease activity indices, can also be reliably captured by PROMs. Previous studies have shown that psychosocial factors, including depression and self‐efficacy, as well as fatigue, are strongly associated with physical function and overall health status in SLE.^8^, ^9^ These domains are increasingly recognized as potential targets for nonpharmacological interventions, particularly to improve daily functioning and quality of life.^10^ Despite this, studies remain sparse and are often conducted in homogenous populations, with limited generalizability and applicability to clinical practice.

The importance of preserving and promoting physical function in SLE has led to the development of expert recommendations on the role of physical activity and structured exercise in SLE, aiming to counteract effects of deconditioning and improve patient outcomes.^11^ However, functional trajectories and the factors that shape them, including psychological factors, remain poorly understood. Recent data from our group suggest substantial differences in physical function between sociodemographically distinct groups, which are not fully explained by ethnic or racial differences, but rather appear to be linked to underlying clinical characteristics and other contextual factors.^12^

To further understand the drivers of perceived physical function in people with SLE, particularly in diverse real‐world settings, we sought to identify modifiable psychological factors that contribute to patient outcomes and inform more equitable and patient‐centered approaches to SLE assessment and management. Specifically, we aimed to investigate the associations of psychosocial factors and fatigue with self‐rated physical function in two demographically distinct SLE cohorts.

## METHODS

### Study population

This was a cross‐sectional study in two distinct cohorts of people with SLE. This cross‐sectional study comprised two sociodemographically distinct US cohorts recruited from populations in Georgia (Approaches to Positive, Patient‐Centered Experiences of Aging with Lupus [APPEAL] and California (California Lupus Epidemiology Study [CLUES]). APPEAL was a cross‐sectional, single‐visit study (October 2019 to May 2022) assessing various geriatric syndromes among adults with SLE^3^ and was ancillary to, and recruited participants from, the ongoing Georgians Organized Against Lupus (GOAL) cohort study. GOAL is a population‐based study of adults (≥18 years) in metropolitan Atlanta with a clinic‐confirmed diagnosis of SLE (four or more revised American College of Rheumatology [ACR] criteria, or three ACR criteria plus a diagnosis of SLE by an attending board‐certified rheumatologist).^13^ GOAL participants are surveyed annually using validated self‐reported instruments to gather data on social determinants of health and disease outcomes.^14^ Inclusion criteria for APPEAL at recruitment were active participation in GOAL, ability to speak English, sufficient vision and hearing to undergo study testing, capacity to consent, and residence in Georgia.

CLUES participants are adults (≥18 years) living in the San Francisco Bay Area with a clinician‐confirmed diagnosis of SLE per the ACR criteria or with confirmed lupus nephritis. CLUES participants are surveyed annually using validated self‐reported instruments to gather data on social determinants and disease outcomes. For this study, data from CLUES participants’ enrollment visits (February 2015 to January 2018) were included.

### Study variables

#### Perceived physical function

To assess physical function, the PROMIS Physical Function Short Forms were used (12a in APPEAL and 10a in CLUES),^7^ with raw scores scaled to comparable T scores. The average T score among adults in the general population is 50 (SD 10), and higher scores denote better perceived physical function. Prior work in rheumatic disease cohorts suggests that a difference of 2 to 3 points in physical function T scores may represent a clinically meaningful change.^15^

#### Fatigue

Fatigue in both APPEAL and CLUES was measured using a single item (“In the past 3 months, how bad has your fatigue been?”) from the Systemic Lupus Erythematosus Questionnaire (SLAQ).^16^ SLAQ items use a 4‐point scale denoted as follows: “no problem,” 0; “mild,” 1; “moderate,” 2; and “severe,” 3.

#### Depression

In APPEAL, depression was assessed using the eight‐item PROMIS depression scale, with scores scaled to T scores, which were categorized to allow comparison with data on depression in CLUES: “none‐slight,” T score <55; “mild,” T score ≥55 and <60; “moderate,” T score ≥60 and ≤70; and “severe,” T score >70. Prior work suggests that a difference of 3 to 4 points in the PROMIS depression T scores represents a clinically meaningful difference in chronic disease populations.^17^

In CLUES, depression was measured using the eight‐item Patient Health Questionnaire (PHQ‐8).^18^, ^19^ The total score ranges from 0 to 24, with higher scores denoting higher levels of depression. The scores were categorized as follows: “none‐minimal,” total score ≤4; “mild,” total score of 5 to 9; “moderate,” total score of 10 to 14; “moderate‐severe,” total score of 15 to 19; and “severe,” total score ≥20. The minimal clinically important difference has been estimated as 5 points, applicable to most chronic disease populations.^20^

#### Perceived stress

Stress was measured using the Perceived Stress Scale (PSS), with the 10‐item version (PSS‐10) used in APPEAL and the 4‐item version (PSS‐4) used in CLUES. Each question is scored 0 (never) to 4 (very often), and the scores are then totaled to give an overall score (range 0–40 for PSS‐10 and 0–16 for PSS‐4). Scores were standardized to z scores to facilitate comparison.

#### Coping

In APPEAL, coping was measured using the 26‐item Coping Self‐Efficacy Scale.^21^ In CLUES, coping was measured using the four‐item Brief Resilient Coping Scale.^22^ Scores from both measures were standardized to yield comparable metrics.

#### General self‐efficacy: CLUES only

Self‐efficacy refers to an individual's belief in their ability to manage symptoms and challenges associated with chronic illness. In our study, general self‐efficacy was measured using the four‐item PROMIS General Self‐Efficacy 4a assessment,^23^, ^24^ with total scores converted to PROMIS T scores. T scores were subsequently categorized as low (<40), average (40–60), or high (≥60) self‐efficacy.

#### Learned helplessness: APPEAL only

Learned helplessness reflects a psychological state in which individuals perceive a lack of control over outcomes, leading to passive coping and disengagement from health care. Here, learned helplessness was scored using the Rheumatology Attitudes Index (RAI)^25^ between 5 and 25, with higher scores indicating greater helplessness. The total score was standardized for this analysis.

#### Other variables

Demographics such as age, sex at birth, race and ethnicity, educational attainment, and income were self‐reported by participants at the study visit. Race and ethnicity were selected from a set of given categories for both cohorts, which were then combined and categorized as Asian, Black, Hispanic, White, and other. Educational attainment was categorized as high school or less, some college or associates degree, or college degree or higher. Income was reported as either low (<$40,000 per annum) or high (≥$40,000 per annum).

Current glucocorticoid and immunosuppressant use at the time of recruitment was recorded. Disease activity and damage were measured using the weighted SLAQ score and the Brief Index of Lupus Damage (BILD), respectively. The SLAQ score is a validated patient‐reported measure of lupus activity, with higher scores denoting greater disease activity.^26^ BILD is a validated patient‐reported instrument that captures cumulative organ damage across multiple systems, with higher scores reflecting greater accumulated damage.^27^

### Statistical analysis

Cohort characteristics were presented using descriptive statistics. Multivariable linear regression models were used to estimate the association between each exposure variable and PROMIS physical function, adjusting sequentially for (1) age and sex; (2) age, sex, and race; (3) age, sex, race, and cumulative disease damage (BILD); and (4) age, sex, race, damage, and disease activity (SLAQ) (except for the association between fatigue and physical function, as fatigue was measured using the SLAQ items). Results were presented as β coefficients with 95% confidence intervals (CIs). Complete case analysis was used. All analyses were conducted using Stata v19.5.

### Ethics

The Emory University Institutional Review Board (IRB) approved the original APPEAL (IRB00110977) and GOAL (IRB00003656) protocols. Analysis of APPEAL data is currently covered by the University of California, San Francisco (UCSF) IRB (23‐39650). All APPEAL participants provided informed consent before completing study visits. The UCSF IRB approved the CLUES study (14‐14429), and all CLUES participants provided informed consent before completing study visits.

## RESULTS

### Cohort characteristics

Overall, 451 participants were included in APPEAL and 580 were included in CLUES. Characteristics of both cohorts are presented in Table 1. The two cohorts were similar in age and sex distribution: mean ages were 46.2 (SD 11.8) and 45.0 (SD 14.5) years in APPEAL and CLUES, respectively, and 91.8% and 90.3% were female. However, APPEAL versus CLUES had a slightly higher percentage of individuals aged 50 years or older (41.0% vs 37.2%) but a lower percentage of individuals aged 65 years or older (4.9% vs 11.2%). Race distribution differed between the two cohorts, with APPEAL being a predominantly Black population (82.5% vs 15.3% in CLUES) and having relatively lower proportions of White, Hispanic, and Asian participants compared to CLUES (11.5% vs 27.4%; 0.2% vs 22.9%; and 1.6% vs 29.3%, respectively). A higher percentage of individuals were college graduates in CLUES (48.8%) compared to APPEAL (38.8%), and a higher percentage of individuals in CLUES reported earning $40,000 or more (59.8%) compared to APPEAL (41.7%).

**Table 1:** Characteristics of included participants from the CLUES and APPEAL cohorts*

Characteristic	APPEAL (n = 451)	CLUES (n = 580)
Sociodemographics		
Age, mean (SD), y	46.2 (11.8)	45.0 (14.5)
18–34	92 (20.4)	173 (29.8)
35–49	174 (38.6)	191 (32.9)
≥50	185 (41.0)	216 (37.2)
Sex at birth, n (%)		
Female	414 (91.8)	524 (90.3)
Male	37 (8.2)	56 (9.7)
Race and ethnicity, n (%)		
Asian	7 (1.6)	170 (29.3)
Black	372 (82.5)	89 (15.3)
Hispanic	1 (0.2)	133 (22.9)
White	52 (11.5)	159 (27.4)
Other	19 (4.2)	29 (5.0)
Education, n (%)		
High school or less	103 (22.8)	117 (20.2)
Some college or associates degree	173 (38.4)	178 (30.7)
College degree or higher	175 (38.8)	283 (48.8)
Missing	0	2 (0.3)
Income, n (%)		
Low (<$40,000)	250 (55.4)	171 (29.5)
High (≥$40,000)	188 (41.7)	347 (59.8)
Missing	13 (2.9)	62 (10.7)
Clinical		
Disease duration, median (IQR), y	14.8 (9.2–21.9)	15 (7–23)
SLAQ score, median (IQR)	11 (6–16)	8 (3–14)
BILD score, median (IQR)	2 (1–4)	1 (0–3)
Current glucocorticoid use, n (%)		
Yes	192 (42.6)	288 (49.7)
No	258 (57.2)	58 (10.0)
Missing	1 (0.2)	234 (40.3)
Immunosuppressant use, n (%)		
Yes	373 (82.7)	480 (82.8)
No	78 (17.3)	22 (3.8)
Missing	0	78 (13.4)
Psychosocial factors and fatigue		
Depression, n (%)		
None/mild	381 (84.4)	432 (74.5)
Moderate/severe	53 (11.8)	138 (23.8)
Missing	17 (3.8)	5 (0.9)
Fatigue, n (%)		
None	49 (10.9)	177 (30.5)
Mild	158 (35.0)	144 (24.8)
Moderate	150 (33.3)	163 (28.1)
Severe	94 (20.8)	91 (15.7)
Missing	0	5 (0.9)
Perceived stress, mean score (SD)^a^	15.2 (7.1)	5.0 (3.3)
Missing, n (%)	36 (8.0)	5 (0.9)
Coping, mean score (SD)	186.8 (56.1)	–
Missing (n, %)	2 (0.4)	–
Self‐efficacy, n (%)		
Low (<40)	–	30 (5.2)
Average (40–60)	–	100 (17.2)
High (>60)	–	19 (3.3)
Missing	–	431 (74.3)
Standardized learned helplessness, mean score (SD)	12.2 (4.6)	–
Missing, n (%)	19 (4.2)	–
Resilience, mean score (SD)	–	8.6 (1.9)
Missing, n (%)	–	432 (74.5)
Physical function		
PROMIS Physical Function 10a/10b T score, mean (SD)	43.6 (9.1)	47.2 (9.8)

* APPEAL, Approaches to Positive, Patient‐Centered Experiences of Aging with Lupus; CLUES, California Lupus Epidemiology Study; IQR, interquartile range; PROMIS, Patient‐Reported Outcomes Measurement Information System; SLAQ, Systemic Lupus Activity Questionnaire; BILD, Brief Index of Lupus Damage.

^a^ Perceived stress was measured using the 10‐item Perceived Stress Scale (PSS‐10) in APPEAL and PSS‐4 in CLUES.

Percentages of participants reporting glucocorticoid and immunosuppressive use at enrollment were similar across cohorts (albeit with a high proportion of missing data in CLUES: 40.3% missing for glucocorticoid use and 13.4% missing for immunosuppressant use). SLAQ scores were higher in APPEAL (median 11, interquartile range [IQR] 6–16) than in CLUES (median 8, IQR 3–14). Damage due to SLE was also greater in APPEAL, with a median BILD score of 3 (IQR 1–4), compared to a median score of 1 (IQR 0–3) in CLUES.

### Factors associated with perceived physical function

PROMIS physical function T scores were slightly higher in CLUES (mean 47.2, SD 9.8) than in APPEAL (mean 43.6, SD 9.1) (Table 1). Results of sequentially adjusted analyses are presented in Supplementary Tables 1 (age and sex adjusted) 2 (age, sex, and race adjusted), 3 (adjusting further for cumulative damage), and 4 (adjusting further for disease activity).

#### Fatigue

In CLUES, the most commonly reported SLAQ score for fatigue was 0 (none; 30.8%), compared to APPEAL, in which the most commonly reported SLAQ score was 1 (mild; 35.0%). The most severe levels of fatigue were reported by 20.8% versus 15.8% of participants in APPEAL versus CLUES, respectively. In both APPEAL and CLUES, higher levels of fatigue were associated with worse physical function, with a greater strength of association observed at all three levels (mild, moderate, severe) in CLUES (Figure 1; Supplementary Table 2). Adjusting for disease damage (Supplementary Table 3) did not overall change the strength of associations between mild or moderate fatigue and physical function in either APPEAL or CLUES. However, in both cohorts, the strength of association between severe fatigue and physical function was reduced (APPEAL: β = −11.03 [95% CI −13.74 to −8.32]; CLUES: β = −12.52 [95% CI −17.60 to −7.43]).

**Figure 1:**
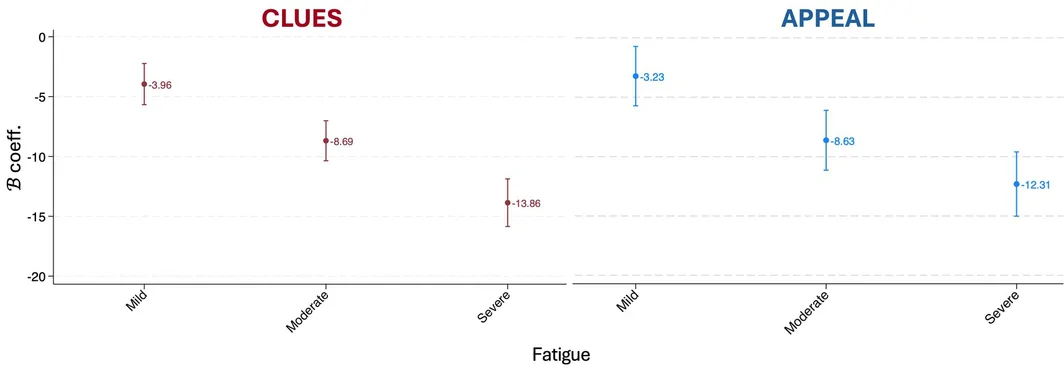
Linear regression analysis, adjusted for age, sex, and race, of the association between fatigue (as measured by a single item from the Systemic Lupus Erythematosus Questionnaire) and self‐perceived physical function for the California Lupus Epidemiology Study (CLUES; left panel) and Approaches to Positive, Patient‐Centered Experiences of Aging with Lupus (APPEAL; right panel).

#### Depression

A greater percentage of participants reported moderate to severe levels of depression in CLUES (24.1% of the total cohort) compared to APPEAL (12.2%). In both cohorts, greater levels of depression were associated with a >1‐SD lower physical function score, albeit with a relatively wide CI for severe depression and function in CLUES (β = −12.65 [95% CI −17.89 to −7.41]) (Figure 2). Adjusting the models for disease damage (Supplementary Table 3) reduced the strength of association between physical function and both mild and moderate‐severe depression in APPEAL, as well as moderate‐severe depression in CLUES, but associations otherwise remained unchanged. However, controlling further for disease activity markedly reduced the strength of association between depression, particularly severe depression, and physical function in both cohorts, albeit remaining statistically significant (Supplementary Table 4).

**Figure 2:**
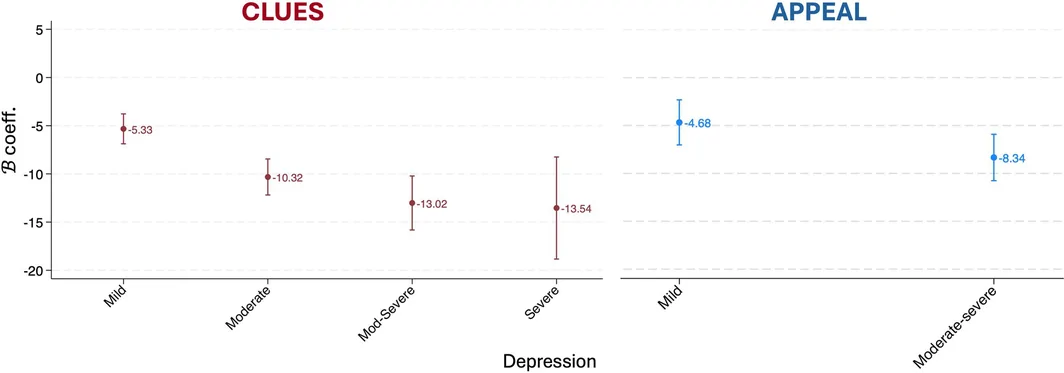
Linear regression analysis, adjusted for age, sex, and race, of the association between depression and self‐perceived physical function for the California Lupus Epidemiology Study (CLUES; left panel) and Approaches to Positive, Patient‐Centered Experiences of Aging with Lupus (APPEAL; right panel). In CLUES, depression was measured using the eight‐item Patient Health Questionnaire. In APPEAL, depression was measured using the eight‐item Patient‐Reported Outcomes Measurement Information System depression scale. Color figure can be viewed in the online issue, which is available at http://onlinelibrary.wiley.com/doi/10.1002/acr.80065/abstract.

#### Perceived stress

The mean PSS‐4 score in CLUES was 5.0 (SD 3.3, range 0–16), whereas the mean PSS‐10 score in APPEAL was 15.2 (SD 7.1, range 0–40). z‐standardized scores in both data sets were close to 0. In both cohorts, worse perceived stress was associated with worse physical function, although the magnitude of association was greater in CLUES (β = −4.26 [95% CI −4.92 to −3.60]) compared to APPEAL (β = −2.87 [95% CI −3.64 to −2.09]). Adjusting the models for disease damage and activity (Supplementary Table 4) reduced the strength of association in both cohorts more than adjusting for damage alone (Supplementary Table 3), but associations remained statistically significant.

#### Coping and resilience

In APPEAL, greater coping efficacy was associated with better self‐reported physical function (β = 3.15 [95% CI 2.39–3.91]). In CLUES, greater resilience was associated with greater levels of physical function (β = 2.04 [95% CI 0.57–3.50]). Adjusting the models further for disease damage (Supplementary Table 3) did not change the strength of associations overall; adjusting further for disease activity did reduce the strength, but the associations remained statistically significant (Supplementary Table 4).

#### Self‐efficacy

Most CLUES participants reported medium levels of self‐efficacy (67.1%). Greater self‐efficacy PROMIS T scores were associated with better physical function in CLUES (average vs low self‐efficacy: β = 6.63 [95% CI 3.13–10.13]; high vs low self‐efficacy: β = 13.53 [95% CI 8.69–18.37]). Adjusting further for both disease damage and activity (Supplementary Table 4) markedly reduced the strength of association between average or high self‐efficacy and worse physical function compared to adjusting for disease damage alone (Supplementary Table 3).

#### Learned helplessness

The mean learned helplessness score in APPEAL, as per the RAI (range 5–25) was 12.2 (SD 4.6), with worse scores associated with poorer self‐reported physical function (β = −3.88 [95% CI −4.62 to −3.14]). Adjustment for disease activity but not disease damage substantially reduced this association (Supplementary Tables 3 and 4).

## DISCUSSION

Our study highlights important associations of psychosocial factors and fatigue with self‐reported perceived physical function in two diverse cohorts of people living with SLE in metropolitan Atlanta and San Francisco. To our knowledge, this is the largest and most diverse SLE cohort in which perceived physical function has been assessed alongside other important clinical, psychological, and quality‐of‐life factors.

Despite demographic differences, particularly in race, greater levels of fatigue, depression, stress, and learned helplessness and worse levels of coping and self‐resilience were all associated with worse perceived physical function in both cohorts. In APPEAL, the strongest association was observed between severe fatigue and physical functioning. In CLUES, the strongest associations were observed for severe levels of depression, self‐efficacy, and fatigue with physical functioning. However, adjustment for perceived disease activity (but not damage) markedly reduced the observed strength of many of these associations.

Although similar overall, the observed differences in the magnitude of associations between cohorts likely reflect differences in the complex interplay of sociodemographic, contextual, and structural influences.^28^ Importantly, given the consistency of results despite sociodemographic differences between the two cohorts, our results are likely generalizable across populations. Whereas previous studies have highlighted racial and ethnic disparities in outcomes in people with SLE, we assessed a range of psychosocial factors, alongside physical functioning, in two demographically distinct cohorts.^29^ Our results indicate that regardless of sociodemographic background, addressing psychosocial factors, as well as disease activity, has the potential to improve physical function and overall well‐being in SLE.

We have reported elsewhere on the differences in perceived physical function between the two cohorts,^12^ with lower self‐reported physical function in APPEAL (mean 43.6) than CLUES (mean 47.2). It is also important to note the differences in the exposures of interest in our study, specifically fatigue, depression, stress, and coping. CLUES participants were more likely than APPEAL participants to have moderate to severe depression (24.1% vs 12.2%). Although it is difficult to estimate the prevalence of depression within populations of people with SLE, estimates suggest a range of 8.7% to 78.6%; the high variability is likely due to a combination of lack of consistency of metrics and definitions of depression in SLE.^30^, ^31^ More strikingly, more than half of APPEAL participants reported moderate‐severe levels of fatigue (54.1%), whereas more than half of CLUES participants reported none/mild fatigue (55.8%). As with depression, estimates for the prevalence of fatigue in people with SLE are highly variable, dependent on population and method of assessment.^32^ Fatigue is also multifactorial; the disparities in fatigue noted between the two cohorts may be due to several reasons.^32^ For example, greater levels of fatigue in APPEAL may reflect greater barriers to health care services, employment, or social support or psychosocial stressors that can often be amplified in underserved populations.^33^, ^34^ Fatigue can also be an indicator of SLE disease activity; greater levels may indicate factors such as structural barriers to care, medication access, or adherence.^35^ The strong association we observed across both cohorts reinforces the role of fatigue as a direct determinant of poor physical function in SLE. Severe fatigue may directly impact mobility and participation in daily activities, thereby reducing patient‐reported physical function. Depression and self‐efficacy were also strongly associated with physical function, especially in CLUES, likely due to more indirect behavioral and cognitive pathways, including reduced motivation and engagement in self‐management. When fatigue burden is high, as observed in APPEAL, it may be the dominant driver of functional limitation, potentially attenuating the relative contribution of the other assessed psychosocial constructs.

Irrespective of cohort, all measures of psychological functioning and fatigue were strongly associated with physical function. This reinforces their importance as consistent and reliable correlates of physical function and their relevance in the clinical setting, particularly in routine monitoring and personalized care and, more broadly, clinical trials.^36^ Assessments of psychological functioning can provide important insights into not only disease activity but also downstream function and outcomes.^37^ Resilience, self‐efficacy, and learned helplessness represent complementary psychosocial constructs that describe how individuals respond to the challenges of stressors such as chronic illness, including SLE. Resilience reflects the capacity to adapt to stress and recover from setbacks. Self‐efficacy refers to confidence in one's ability to manage symptoms and daily activities and has been linked to other psychosocial factors such as fatigue, depression, and pain.^38^ In contrast, learned helplessness reflects a perceived lack of control over health outcomes and is associated with passive coping and reduced activity. Although not routinely measured in rheumatology practice, these constructs may influence how patients interpret symptoms and adhere to treatment.

Crucially, the exposures examined here were more strongly associated with physical function than some routinely collected data, such as disability benefits receipt.^39^ Our results highlight potentially modifiable psychosocial factors that may subsequently impact patient outcomes, such as mental health symptoms and stress. Such factors have previously been demonstrated as being associated with worse disease activity in SLE, including within CLUES; patients who experienced greater stress had significantly worse disease activity and greater symptom burden at follow‐up compared to those with stable or declining stress levels.^40^ Mental health conditions such as depression and anxiety parallel changes in patients’ self‐assessment of disease activity and increase the risk of experiencing suboptimal health and functional outcomes.^41^ Psychosocial needs of people living with SLE remain largely unmet; our results add to the growing body of evidence that addressing psychosocial factors may potentially improve overall physical well‐being in this population.^42^

Additionally, the strong associations observed between all measures of psychological function and fatigue and physical function suggest that it may still be possible to gain an understanding of an individual's physical function and overall state of health with a few brief measures. This is important in a busy clinical environment in which both physicians and patients may not have the time or means to complete multiple questionnaires. Brief PROMs assessing factors such as fatigue, depression, stress, and self‐efficacy could be feasibly integrated into routine rheumatology care using short forms (eg, PROMIS instruments, PHQ‐8) administered before clinic visits. Scores could be used to identify patients at risk of poor functional outcomes, prompting targeted interventions such as mental health referral, behavioral interventions, or self‐management support.^37^ Longitudinal monitoring of these measures may also help track response to treatment or nonpharmacological interventions. In clinical trials, systematic collection of these outcomes may improve identification of patient‐centered benefits beyond traditional disease activity measures.^43^ Our results reinforce recommendations from the ACR to assess depression and physical function at least annually in all people with SLE, with the option of adding cognition and fatigue assessments.^37^ In our study, the strongest predictor of physical function across both cohorts was depression, which emphasizes the need to consider both in the clinical setting.

It is important to note the role of cumulative organ damage and ongoing disease activity on our observed associations. Adjustment for cumulative organ damage had relatively little impact on the strength of associations between psychosocial factors and physical function, whereas further adjustment for self‐reported disease activity substantially attenuated them. These observations suggest that current disease burden may play a more prominent role in shaping perceived physical function than accumulated irreversible damage.^44^ The SLAQ score captures symptoms such as fatigue, pain, and cognitive function, which directly influence quality of life and psychosocial well‐being. Conversely, organ damage reflects longer‐term changes, which may be less closely aligned with daily function. The attenuations observed with disease activity may also indicate shared pathways with psychosocial factors influencing physical functioning.

Our observed associations are likely bidirectional. Physical function affects all aspects of a person's life, including work, social participation, and personal relationships.^45^ In our study, worse stress levels and depression were strongly associated with poorer perceived physical function. Data across chronic diseases have demonstrated the benefits of physical exercise for mental health, especially in improving symptoms of depression, regardless of factors such as comorbidities.^46^ Limitations in physical functioning have detrimental impacts on the ability to participate in physical activity, including exercise. Similarly, lower fatigue levels are seen in people with SLE who engage in physical activity.^47^ Self‐efficacy and coping have both previously been reported to be strongly linked with physical symptoms and functioning in lupus, as well as fatigue, indicating the associations are likely to be complex and mediated by multiple factors.^48^ An intervention to enhance behavioral and cognitive coping strategies has led to meaningful improvements in physical and psychological outcomes, suggesting not only that it is worthwhile to assess these parameters in clinic but also that they are potential targets for nonpharmacological interventions to improve quality of life in people with SLE.^49^ Conversely, more routine measurement of patient‐reported outcomes (PROs), especially those that predict physical function, can enable clinicians to develop interventions that align with patients’ priorities, including coping and self‐efficacy. Our results, which identify several distinct drivers of physical function, also highlight the benefits of multidisciplinary care in the management of SLE to address physical function and overall well‐being.

Our study has several limitations. This was a cross‐sectional observational study, and we therefore cannot infer causality from the results of our analyses. We were also unable to assess changes in either the exposure or the outcome variable over time, which is important to consider in the setting of a fluctuant disease state, such as SLE. Specifically, for APPEAL, data collection occurred during the COVID‐19 pandemic, a period associated with substantial changes in health care use, psychosocial stress, and daily functioning, especially for people with SLE. We are unable to account for potential temporal effects, which may have influenced PROs. The two cohorts used different measures to assess depression, stress, coping, self‐efficacy, and learned helplessness. Values were either categorized or standardized to facilitate comparisons between groups. Additionally, both cohorts were based in the United States, and associations between psychosocial factors and physical function may differ in other geographic or health care contexts. Variations in aspects such as health care access, cultural perceptions of health, and availability of multidisciplinary resources could influence both the prevalence of these factors and their impact on functional outcomes. Future studies in international and more diverse populations are needed to determine the broader applicability of these findings.

However, our study is strengthened by the use of two large sociodemographically diverse cohorts of people with SLE from two distinct US populations. This in turn supports the generalizability of our findings and the need to assess PROs and consider a patient's perceived physical function.

In conclusion, in this study of two sociodemographically distinct cohorts of people with SLE, we found that PROs, including fatigue, depression, stress, coping, and self‐efficacy, were consistently and strongly associated with perceived physical function. Despite marked differences in cohort composition, these associations were observed in both cohorts, highlighting the central role of psychosocial and subjective experiences in determining functional outcomes in SLE. Our findings reinforce the importance of routinely incorporating PROs into clinical practice, not only to capture patient experiences but also to inform prognostic assessment and guide holistic, patient‐centered care.

## AUTHOR CONTRIBUTIONS

All authors contributed to at least one of the following manuscript preparation roles: conceptualization AND/OR methodology, software, investigation, formal analysis, data curation, visualization, and validation AND drafting or reviewing/editing the final draft. As corresponding author, Dr Dey confirms that all authors have provided the final approval of the version to be published and takes responsibility for the affirmations regarding article submission (eg, not under consideration by another journal), the integrity of the data presented, and the statements regarding compliance with institutional review board/Declaration of Helsinki requirements.

## Supporting information


**Disclosure Form**:

**Supplementary Table 1.** Associations of patient‐reported outcomes with physical function, adjusting for age and sex.

**Supplementary Table 2.** Associations of patient‐reported outcomes with physical function, adjusting for age, sex and race.

**Supplementary Table 3.** Associations of patient‐reported outcomes with physical function, adjusting for age, sex, race and damage (as measured by BILD).

**Supplementary Table 4.** Associations of patient‐reported outcomes with physical function, adjusting for age, sex, race, damage (as measured by BILD) and disease activity (as measured by SLAQ).
