# Supplementary material for: The Association of Physical Function With Psychosocial Patient‐Reported Outcomes in People With Systemic Lupus Erythematosus
Source: Arthritis Care Res (Hoboken). 2026 May 3;78(11):1631–9. doi: 10.1002/acr.80065 (PMC13710787; doi:10.1002/acr.80065)
Supplement: Supplementary file 2 [file ACR-78-1631-s002.docx]

**Supplementary Table 1: Associations of patient-reported outcomes with physical function, adjusting for age and sex.**

| **CLUES (Total n=580)** | | | |
| --- | --- | --- | --- |
| Exposure variable | n | ß coefficient | 95% CI |
| Depression (PHQ-8)  None-minimal (0-4)  Mild (5-9)  Moderate (10-14)  Moderately severe (15-19)  Severe (≥20) | Total=575  272 (48.2)  160 (27.8)  94 (16.4)  35 (6.1)  9 (1.6) | Referent  -5.33  -10.32  -13.02  -13.54 | -  -6.88, -3.77  -12.19, -8.45  -15.82, -10.22  -18.84, -8.24 |
| Fatigue (SLAQ item)  None (0)  Mild (1)  Moderate (2)  Severe (3) | Total=575  177 (30.8)  144 (25.0)  163 (28.4)  91 (15.8) | Referent  -4.08  -9.01  -14.08 | Referent  -5.79, -2.37  -10.67, -7.34  -16.07, -12.10 |
| Self-efficacy (PROMIS T-score)  Low (<40)  Average/ Medium (40-60)  High (>60) | Total=149  30 (20.1)  100 (67.1)  19 (12.8) | Referent  7.02  13.11 | -  3.55, 10.48  8.28, 17.94 |
| Resilience (standardised) | 148 | 1.96 | 0.49, 3.43 |
| Stress (standardised) | 575 | -4.34 | -5.00, -3.67 |
|  | | | |
| **APPEAL (Total n=451)** | | | |
| Depression (PROMIS T-score)  None-slight (<55)  Mild (55-59)  Moderate-severe (≥60) | Total=434  324 (74.7)  57 (13.1)  53 (12.2) | Referent  -4.45  -8.54 | -  -6.82, -2.07  -10.99, -6.09 |
| Fatigue (SLAQ item)  None (0)  Mild (1)  Moderate (2)  Severe (3) | Total=451  49 (10.9)  158 (35.0)  150 (33.3)  94 (20.8) | Referent  -3.01  -8.41  -12.17 | -  -5.55, -0.47  -10.97, -5.85  -14.91, -9.43 |
| Coping (standardised corrected CSES) | 449 | 3.08 | 2.30, 3.85 |
| Learned helplessness (standardised) | 432 | -4.02 | -4.75, -3.28 |
| Stress (standardised PSS-10) | 415 | -2.94 | -3.72, -2.15 |
