# Supplementary material for: The Association of Physical Function With Psychosocial Patient‐Reported Outcomes in People With Systemic Lupus Erythematosus
Source: Arthritis Care Res (Hoboken). 2026 May 3;78(11):1631–9. doi: 10.1002/acr.80065 (PMC13710787; doi:10.1002/acr.80065)
Supplement: Supplementary file 3 [file ACR-78-1631-s003.docx]

**Supplementary Table 2: Associations of patient-reported outcomes with physical function, adjusting for age, sex and race.**

| **CLUES (Total n=580)** | | | |
| --- | --- | --- | --- |
| Exposure variable | n | ß coefficient | 95% CI |
| Depression (PHQ-8)  None-minimal (0-4)  Mild (5-9)  Moderate (10-14)  Moderately severe (15-19)  Severe (≥20) | Total=575  272 (48.2)  160 (27.8)  94 (16.4)  35 (6.1)  9 (1.6) | Referent  -5.15  -10.21  -13.05  -12.65 | -  -6.68, -3.62  -12.06, -8.36  -15.81, -10.28  -17.89, -7.41 |
| Fatigue (SLAQ item)  None (0)  Mild (1)  Moderate (2)  Severe (3) | Total=575  177 (30.8)  144 (25.0)  163 (28.4)  91 (15.8) | Referent  -3.96  -8.69  -13.86 | Referent  -5.67, -2.24  -10.36, -7.02  -15.85, -11.87 |
| Self-efficacy (PROMIS T-score)  Low (<40)  Average/ Medium (40-60)  High (>60) | Total=149  30 (20.1)  100 (67.1)  19 (12.8) | Referent  6.63  13.53 | -  3.13, 10.13  8.69, 18.37 |
| Resilience (standardised) | 148 | 2.04 | 0.57, 3.50 |
| Stress (standardised) | 575 | -4.26 | -4.92, -3.60 |
|  | | | |
| **APPEAL (Total n=451)** | | | |
| Depression (PROMIS T-score)  None-slight (<55)  Mild (55-59)  Moderate-severe (≥60) | Total=434  324 (74.7)  57 (13.1)  53 (12.2) | Referent  -4.68  -8.34 | -  -7.03, -2.33  -10.76, -5.92 |
| Fatigue (SLAQ item)  None (0)  Mild (1)  Moderate (2)  Severe (3) | Total=451  49 (10.9)  158 (35.0)  150 (33.3)  94 (20.8) | Referent  -3.23  -8.63  -12.32 | -  -5.73, -0.73  -11.15, -6.11  -15.02, -9.61 |
| Coping (standardised corrected CSES) | 449 | 3.15 | 2.39, 3.91 |
| Learned helplessness (standardised) | 432 | -3.88 | -4.62, -3.14 |
| Stress (standardised PSS-10) | 415 | -2.87 | -3.64, -2.09 |
