# Supplementary material for: The Association of Physical Function With Psychosocial Patient‐Reported Outcomes in People With Systemic Lupus Erythematosus
Source: Arthritis Care Res (Hoboken). 2026 May 3;78(11):1631–9. doi: 10.1002/acr.80065 (PMC13710787; doi:10.1002/acr.80065)
Supplement: Supplementary file 4 [file ACR-78-1631-s004.docx]

**Supplementary Table 4: Associations of patient-reported outcomes with physical function, adjusting for age, sex, race, damage (as measured by BILD) and disease activity (as measured by SLAQ).**

| **CLUES (Total n=580)** | | | |
| --- | --- | --- | --- |
| Exposure variable | n | ß coefficient | 95% CI |
| Depression (PHQ-8)  None-minimal (0-4)  Mild (5-9)  Moderate (10-14)  Moderately severe (15-19)  Severe (≥20) | Total=575  272 (48.2)  160 (27.8)  94 (16.4)  35 (6.1)  9 (1.6) | Referent  -2.37  -4.67  -5.47  -5.16 | -  -3.84, -0.90  -6.63, -2.72  -8.29, -2.64  -10.10, -0.23 |
| Self-efficacy (PROMIS T-score)  Low (<40)  Average/ Medium (40-60)  High (>60) | Total=149  30 (20.1)  100 (67.1)  19 (12.8) | Referent  1.09  5.71 | -  -2.29, 4.48  0.97, 10.45 |
| Resilience (standardised) | 148 | 0.29 | -1.03, 1.61 |
| Stress (standardised) | 575 | -2.01 | -2.68, -1.34 |
|  | | | |
| **APPEAL (Total n=451)** | | | |
| Depression (PROMIS T-score)  None-slight (<55)  Mild (55-59)  Moderate-severe (≥60) | Total=434  324 (74.7)  57 (13.1)  53 (12.2) | Referent  -1.43  -2.91 | -  -3.50, 0.63  -5.16, -0.66 |
| Coping (standardised corrected CSES) | 449 | 1.08 | 0.37, 1.79 |
| Learned helplessness (standardised) | 432 | -1.89 | -2.63, -1.15 |
| Stress (standardised PSS-10) | 415 | -0.95 | -1.66, -0.24 |

BILD: Brief Index of Lupus Damage. SLAQ: Systemic Lupus Activity Questionnaire.

Fatigue not included in analyses due to being measured as a subset of the overall SLAQ score.
