# Supplementary material for: The Association of Physical Function With Psychosocial Patient‐Reported Outcomes in People With Systemic Lupus Erythematosus
Source: Arthritis Care Res (Hoboken). 2026 May 3;78(11):1631–9. doi: 10.1002/acr.80065 (PMC13710787; doi:10.1002/acr.80065)
Supplement: Supplementary file 5 [file ACR-78-1631-s005.docx]

**Supplementary Table 3: Associations of patient-reported outcomes with physical function, adjusting for age, sex, race and damage (as measured by BILD).**

| **CLUES (Total n=580)** | | | |
| --- | --- | --- | --- |
| Exposure variable | n | ß coefficient | 95% CI |
| Depression (PHQ-8)  None-minimal (0-4)  Mild (5-9)  Moderate (10-14)  Moderately severe (15-19)  Severe (≥20) | Total=575  272 (48.2)  160 (27.8)  94 (16.4)  35 (6.1)  9 (1.6) | Referent  -4.93  -9.70  -12.06  -12.52 | -  -6.42, -3.44  -11.50, -7.90  -14.76, -9.36  -17.60, -7.43 |
| Fatigue (SLAQ item)  None (0)  Mild (1)  Moderate (2)  Severe (3) | Total=575  177 (30.8)  144 (25.0)  163 (28.4)  91 (15.8) | Referent  -3.61  -8.27  -12.72 | Referent  -5.30, -1.93  -9.92, -6.63  -14.71, -10.73 |
| Self-efficacy (PROMIS T-score)  Low (<40)  Average/ Medium (40-60)  High (>60) | Total=149  30 (20.1)  100 (67.1)  19 (12.8) | Referent  6.06  12.44 | -  2.55, 9.56  7.50, 17.38 |
| Resilience (standardised) | 148 | 2.16 | 0.72, 3.59 |
| Stress (standardised) | 575 | -3.96 | -4.61, -3.30 |
|  | | | |
| **APPEAL (Total n=451)** | | | |
| Depression (PROMIS T-score)  None-slight (<55)  Mild (55-59)  Moderate-severe (≥60) | Total=434  324 (74.7)  57 (13.1)  53 (12.2) | Referent  -3.54  -7.79 | -  -5.84, -1.23  -10.13, -5.45 |
| Fatigue (SLAQ item)  None (0)  Mild (1)  Moderate (2)  Severe (3) | Total=451  49 (10.9)  158 (35.0)  150 (33.3)  94 (20.8) | Referent  -3.06  -8.22  -11.03 | -  -5.51, -0.61  -10.70, -5.75  -13.74, -8.32 |
| Coping (standardised corrected CSES) | 449 | 2.83 | 2.09, 3.58 |
| Learned helplessness (standardised) | 432 | -3.55 | -4.27, -2.82 |
| Stress (standardised PSS-10) | 415 | -2.67 | -3.42, -1.93 |

BILD: Brief Index of Lupus Damage.
